# Alkali Activation of Metallurgical Slags: Reactivity, Chemical Behavior, and Environmental Assessment

**DOI:** 10.3390/ma14030639

**Published:** 2021-01-30

**Authors:** Isabella Lancellotti, Federica Piccolo, Katja Traven, Mark Češnovar, Vilma Ducman, Cristina Leonelli

**Affiliations:** 1 Department of Engineering ‘Enzo Ferrari’, University of Modena and Reggio Emilia, Via Vivarelli 10, 41125 Modena, Italy; federica.piccolo@unimore.it (F.P.); cristina.leonelli@unimore.it (C.L.); 2 Slovenian National Building and Civil Engineering Institute-ZAG, Dimičeva 12, 1000 Ljubljana, Slovenia; katja.traven@zag.si (K.T.); mark.cesnovar@zag.si (M.Č.); Vilma.Ducman@zag.si (V.D.)

**Keywords:** slag, aluminosilicate materials, chemical reactivity, cold consolidation, alkali activation, leaching tests

## Abstract

Alkali-activated materials (AAMs) represent a promising alternative to conventional building materials and ceramics. Being produced in large amounts as aluminosilicate-rich secondary products, such as slags, they can be utilized for the formulation of AAMs. Slags are partially crystalline metallurgical residues produced during the high temperature separation of metallic and non-metallic materials in the steelmaking processes. In the present study, the electric arc furnace carbon or stainless steel slag (EAF) and secondary metallurgical slag such as ladle furnace basic slag (LS) were used as precursors in an alkali-activation process. EAF slag, with its amorphous fraction of about 56%, presented higher contents of soluble Si and Al species with respect to ladle slag R (35%). However, both are suitable to produce AAM. The leaching behavior shows that all the release values are below the regulation limit. All the bivalent ions (Ba, Cd, Cu, Ni, Pb, and Zn) are well immobilized in a geopolymeric matrix, while amphoteric elements, such as As and Cr, show a slight increase of release with respect to the corresponding slag in alkaline and aqueous environments. In particular, for Sb and As of AAM, release still remains below the regulation limits, while Mo presents an increase of leaching values that slightly exceeds the limit for landfill non-dangerous waste.

## 1. Introduction

Inorganic aluminosilicate materials, also known as alkali-activated materials (AAMs), represent a promising alternative to conventional building materials and ceramics. Large amounts of aluminosilicate-rich secondary products such as fly ashes, volcanic ashes, clays, and slags can be utilized as well [1]. Slags are partially crystalline metallurgical residues produced during the high temperature separation of metallic and non-metallic materials in the steelmaking processes [2]. Due to their mineralogical composition these industrial by-products should be recycled and reused for the economic recovery of the original materials. Several kinds of slags obtained from the different metallurgical processes can be listed:

i. Blast furnace (BF) iron slag, also known as a ground granulated blast furnace slag (GGBFS);
ii. Electric arc furnace carbon or stainless steel slag (EAF-C/S);
iii. Secondary metallurgical slag such as ladle furnace basic slag (LS), also called white slag;
iv. Basic oxygen furnace slag (BOS);
v. Others (e.g., desulphurization slag).

Considerable amounts of about-mentioned slags are already in use as precursors in alkali-activation processes [3,4,5,6].

In particular, Muhmood et al. studied how the replacement of cement by 15 to 30% of EAFS affects the compressive strength of the material. It turned out that the compressive strength was decreased at initial stages (first 14 days). After 28 days, the addition of AEFS contributed to the higher compressive strength in comparison to mortars without EAFS addition [7]. EAFS was also studied by Ozturk et al. in an extensive study where different influential parameters (such as silicate modulus, sodium concentration, relative humidity, curing temperature and curing time) on an alkali-activation process were varied. The best performance was shown in the mortar specimen reaching the compressive and flexural strengths of 22.0 and 4.2 MPa, respectively [8]. In another study, GGBFS-based alkali-activated samples were exposed to heat curing, and the mechanism and microstructure of the alkali-activation process was investigated [9]. Steel slag may also be exploited in cement and concrete production as a raw material for clinker manufacturing and other areas such as sintering additives, heavy metals adsorbents, and asphaltic mixtures etc., [10].

Ladle slag has been investigated for their potential utilization in various applications [11] in conventional concrete [12,13,14]. Ladle slag is used in concrete with polypropylene fibers [15], because the brittleness of the material limits its use in construction, therefore, in this experimental investigation, fibers were employed as a short random reinforcement in cementitious matrix in order to improve the mechanical performance. Ladle slag is also used as a binder in alkali-activated composites [16], mixed with fly ash [17,18], or with metakaolin [19]. Further, ladle furnace slag containing traces of heavy metals was activated with sodium hydroxide (solid NaOH), sodium sulfate (Na_2_SO_4_), and sodium metasilicate (Na_2_SiO_3_) and then blended with ground granulated blast-furnace slag in order to immobilize metals, the results show that ladle slag can be used as a binding material for some civil engineering applications with relatively low requirement on strength and curing time, e.g., soil stabilization [20].

In the present study, the EAF and LS were used as aluminosilicate precursors in alkali-activation process. The activating solution, commercial Na silicate, was adopted to maintain a cost-efficient process. The novelty of this paper consists in the study of reactivity, chemical behavior, and environmental assessment of slag’s pre- and post-alkali activation in order to evaluate the suitability of slags for the formulation of AAMs and their behavior in alkaline environment. Also, how the chemical and mineralogical differences between the slags can influence the chemical performance of the final AAMs is investigated.

## 2. Materials and Methods

### 2.1. Raw Materials

Two different locally available slags were used in this investigation. Slag A is an electric arc furnace slag (from SIJ Acroni, Jesenice, Slovenia). Slag R is a ladle furnace slag (from SIJ Ravne, Ravne na Koroškem, Slovenia), which is obtained within the second stage of refining (during desulfurization). Their chemical analysis, performed by X-ray fluorescence (XRF) with a wavelength dispersive X-ray fluorescence (WD XRF) instrument (Thermo Scientific, Thermo electron SA, Ecublens, Switzerland) is given in Table 1, where the loss on ignition, L.O.I., is also reported. L.O.I. is higher in Slag R, due to its higher amount of carbonates with respect to Slag A (see Table 2 for confirmation). Furthermore, the SiO_2_ + Al_2_O_3_ content is equal to 29.59 and 18.89 wt.% for Slag A and R, respectively, allowing us to hypothesize that Slag A can be more adequate for alkali activation. From Table 1b the presence of small amounts of heavy metals, sulfur and phosphorous are evident.

**Figure 1:**
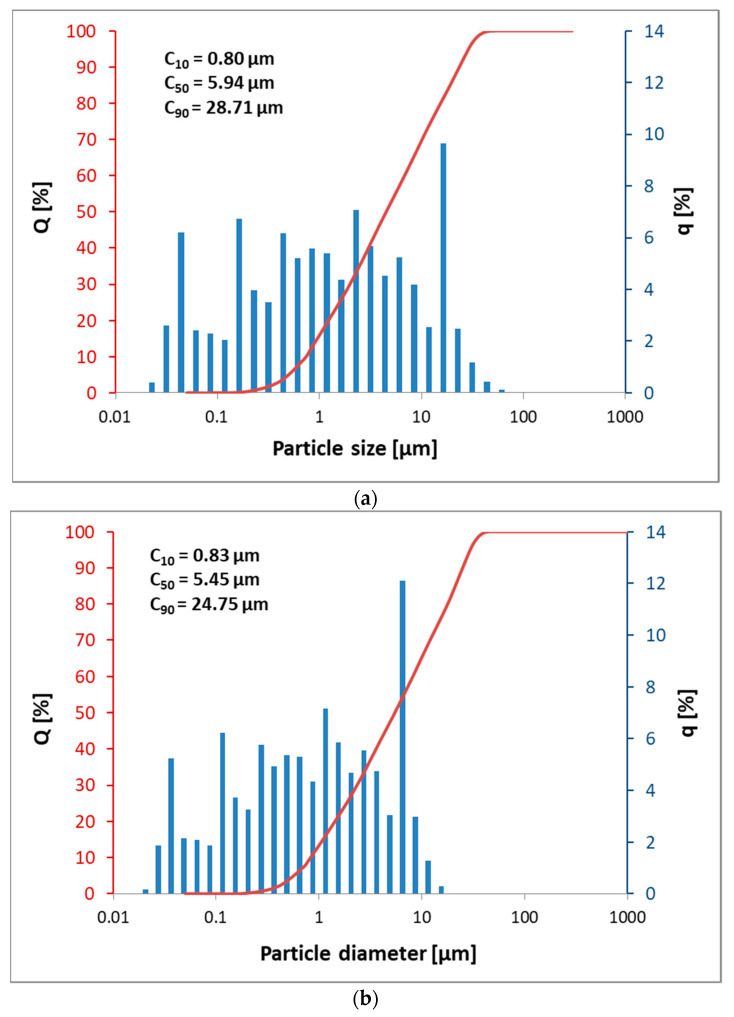
Particle size distribution plotted as cumulative curve (line, red axis) and particle size distribution curve (histogram, blue axis) together with cumulative values at 10, 50, and 90% (C_10_, C_50_ in C_90_) for Slag A (**a**) and Slag R (**b**).

The mineral composition and quantitative determination of mineral phases were based on XRD analysis (X-ray powder diffractometer Malvern PANalytical Empyrean, Surrey, UK) with CuKα radiation λ = 1.54 Å, 2θ range 4°–70°, rate of 0.026°/min and Rietveld refining method with alumina powder as an external standard reference material (NIST 676a) (X’Pert High Score Plus diffraction software with goodness of fit of 4.9). This technique has confirmed that Slag R contains a higher content of calcite (CaCO_3_) and dolomite (MgCa(CO_3_)_2_), 32.6%, with respect to Slag A, 15.8%. These phases are theoretically responsible for the loss of ignition of 15.05% for Slag R and of 7.3% for Slag A. The difference in LOI is due to humidity and organic content. In the context of alkali activation, it can be noted that Slag A contains approx. 56% of amorphous phase, while Slag R contains only 35%. Besides calcite, dolomite, and amorphous phase, both slags contain also quartz (SiO_2_), merwinite ((Ca_3_Mg(SiO_4_)_2_), periclase (MgO), ankerite (Ca(Fe,Mg,Mn)(CO_3_)_2_), corundum (Al_2_O_3_), and wüstite (FeO) and low amount of other phases as reported in Table 2 [21]. The particle size distribution (CILAS 920 (Cilas, Orleans Cedex, France)) was obtained by milling and sieving (63 µm) slag powders and dispersing with microscan dispersant type C (Quantachrome Corporation, FL, USA). Analysis shows the very similar nature of the two slags with very small dimensions of particles, being C90 less than 30 µm (Figure 1).

Microstructural analysis of the hardened AAM fresh fractures was performed by a JEOL scanning electron microscope (SEM) in a back-scattered electrons image mode (JSM-IT500 LV, Jeol, Tokyo, Japan) and in a low vacuum. Quantitative analysis was performed using energy dispersive spectroscopy-EDS (Oxford instruments, Abingdon, UK) using Aztec software platform.

### 2.2. Preparation and Characterization of Alkali-Activated Mixture

The development of different alkali-activated materials based on both slags activated with two different types of activators (sodium- and potassium-based) has already been studied in our previously published papers [21,22]. It has been found that in the case of sodium-based activators, the ideal activator/slag ratio was 0.35, resulting in compressive strengths of 40.3 and 63.2 MPa and bending strengths of 3.8 and 3.6 MPa for Slag A and Slag R, respectively. Therefore, in this study both slags (100 g) were activated by addition of 55 g of sodium silicate (Na_2_SiO_3_) Crystal 0112 produced by Tennants distribution, Ltd. (UK), and additional amount of 8 or 5 g of water for slag A and slag R, respectively. Specifications of sodium glass are as follows: solid content is 48.5%, and mass ratio of Na_2_O to SiO_2_ is 0.51 (Na_2_O = 15.4%, SiO_2_ = 30.4%). Samples are named as A50NW and R50NW. Mixtures were poured in silicon molds and prisms were cured at 70 °C for 3 days. The curing regime was also already optimized and reported by Češnovar et al. [21]. Part of the samples was then used for mechanical properties determination and microstructural analysis, while the other part was used for leaching tests.

**Figure 2:**
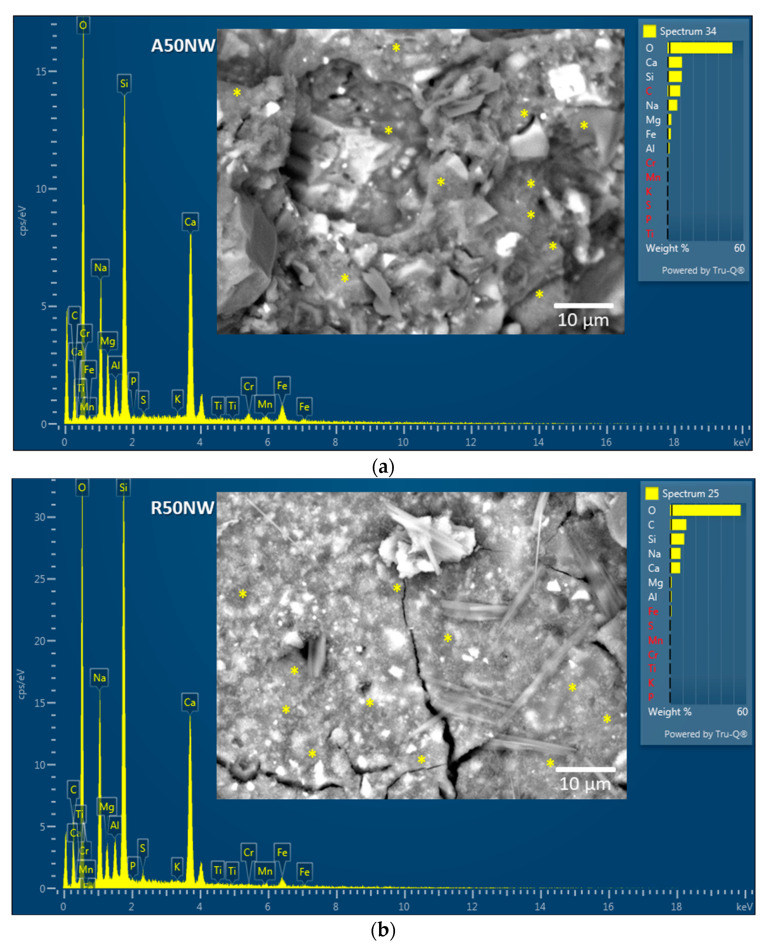
SEM micrographs together with the representative EDS analysis of alkali-activated materials (AAM) matrix for sample A50NW (**a**) and R50NW (**b**); the spots where EDS analysis was taken are marked with yellow stars.

Additionally to analysis, already presented in the above mentioned papers, also microstructural analysis by SEM/EDS on the fresh fractures of samples A50NW and R50NW was carried out (Figure 2a,b). Further in these micrographs, there are some particles after alkali activation (bright spots corresponding to Fe and Cr particles) indicating that they did not react with alkali. Therefore, they present only the filling in the AAM matrix. However, the majority of area in both cases represents an alkali-activated matrix, where element in abundance is Si, followed by Na and Ca. The spots where EDS analysis was taken are marked with yellow stars. On micrograph of sample R50NW (Figure 2b) also some crystals belonging to the precipitated NaCl are observed.

### 2.3. Reactivity and Chemical Behavior of Slags and Alkali Activated Materials

In order to assess the chemical resistance of the alkali-activated materials and their stability in an aqueous environment, integrity test was performed. For the procedure a sample of 3–4 g was immersed in distilled water (solid/liquid ratio wt.% of 1:100) for 24 h at room temperature [23]. The compactness of alkali-activated materials in water confirmed the occurring of efficient consolidation reaction after at least 28 days of aging, i.e., when microstructure reaches stability [24].

The evaluation of the hazardous or not hazardous nature of the slags and the corresponding alkaline-activated materials were assessed from chemical point of view according to the European norm EN 12457-2 [25]. This experimental procedure was also performed on the as-received slags with the aim to compare the amount of released heavy metals with the eluates of same tests performed on AAM. It has been already proved that the alkaline environment typical of AAM fosters the leaching of some elements such as Cr, Ni, As, Cd [26,27]. Ten grams of slag with grain size lower than 4 mm was added to distilled water in a Teflon^®^ bottle with solid/liquid ratio wt.% of 1:10. The mixture was stirred on a magnetic stirrer for 24 h at room temperature, then it was filtered to separate the solid from the liquid fraction. The liquid fraction was acidified at pH 2 with nitric acid for ICP/AES analyses (VARIAN LIBERTY AX) to determine the amount of heavy metals released. During the leaching test, the pH (pH sensor Hamilton type Liq-glass SL) and ionic conductivity (OAKTON Eutech Instruments CON 6/TDS 6) were measured in the leachate after 0, 5, 15, 30, 60, 120, 240, 480, 1440 min from immersion to determine the amount of ions released in solution and define which raw material was best suited to the realization of alkaline activation materials [28]. pH and conductivity measurements were performed also on alkali-activated materials to assess their consolidation process and their three dimensional reticulation.

The quantification of reactive fraction of the slags in strong alkaline environment was evaluated through the basic attack in accordance with the test reported in literature [29]. The experimental procedure consisted in adding one gram of slag in 100 mL of 8 M NaOH solution and stirred constantly for 5 h in a Teflon^®^ bottle at 80 ± 2 °C. The grain size of the slag’s sample was less than 45 μm. The final solution was filtered; the residue was washed with de-ionized water to a neutral pH. The liquid fraction was acidified at pH 2 with nitric acid in order to quantify the contents of Al and Si with ICP/OES (VARIAN LIBERTY AX). The values of Si and Al were used to determine the ratio (SiO_2_/Al_2_O_3_)_reactive_ of each starting material that was fundamental to define at which extend the raw material was suitable for alkaline activation. The insoluble fraction resulting from this test was characterized with XRD in order to estimate the change of crystalline phase after the alkali attack. This test was performed by a powder diffractometer (PW 3710, Philips) with Cu Kα radiation in the 5–70° 2θ range on powdered samples characterized by grain size of 20–30 μm.

In order to evaluate the amount of slag that had been converted to a 3D stable aluminosilicate network and the portion that had not reacted with the alkali solution, acid attack was carried out according to the test reported in literature [30]. This experimental procedure was performed also on slags to estimate the fraction that can be dissolved in acid environment. These results were a reference to interpret the behavior of slags in the alkali-activated materials. One gram of slag was placed in 250 mL of (1:20) HCl (Sigma Aldrich, 37%) and stirred constantly for three hours on a magnetic stirrer at room temperature. The grain size of test sample was less than 1 mm. The resulting solution was then filtered, the insoluble fraction washed with de-ionized water to a neutral pH, then dried at 105 °C and weighed. The degree of reaction was found by determining the weight loss. The same procedure was repeated at least three times to ensure reproducibility. The insoluble residue obtained by acid attack was analyzed by XRD in order to evaluate the modification of crystalline phases after HCl test. This test was carried out by a powder diffractometer (PW 3710). Philips) with Cu Kα radiation in the 5–70° 2θ range on powdered samples.

## 3. Results and Discussion

### 3.1. Slag A and Slag R Characterization

A leaching test according to the European norm EN 12457-2 was necessary to verify the hazardous nature of Slag A and Slag R. The percentages of heavy metals, such as Ba, Pb, Cu, found in the eluate were compared to the limit values prescribed to dispose of the non-dangerous waste in a landfill (Table 3). The legal limit values proposed in Table 4 are those of Italian DM 27/09/2010 which was transposed by European Directive 1999/31/CE (Slovenian limits are the same). The amount of heavy metals was below the regulation limits, so the leaching test confirmed the non-hazardous nature of both the slags and the possibility of using these slags as raw materials to prepare alkali-activated materials.

**Figure 3:**
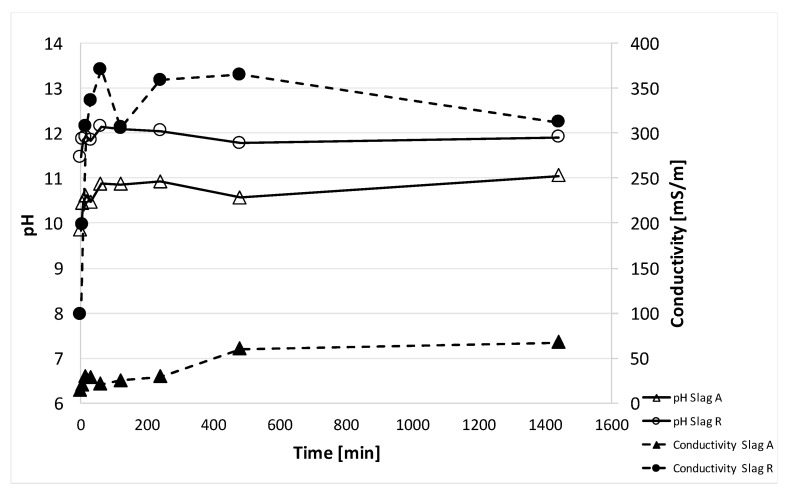
pH and ionic conductivity values of Slag A and Slag R (lines are drawn for eye help, only). The confidence interval for pH is about 1%, while for conductivity is around 2%.

In order to evaluate the release of ions in the eluate of slag A and slag R and their chemical stability in aqueous environment, pH and ionic conductivity measurements were collected at different times: 0, 5, 15, 30, 60, 120, 240, 480, 1440 min after immersion in water (Figure 3). For both slags, pH values around 10–11 remained constant during the first 24 h. The conductivity values of Slag R were not constant, increasing very rapidly in the first 2 or 3 h to reach the value of approx. 300 mS/m within the first 24 h. The sharp increase in ionic conductivity is a consequence of the release of ions in the environment from part of Slag R. Slag A showed lower pH and conductivity with respect to Slag R, so it meant that slag A was more stable and less soluble in aqueous environment than Slag R.

The alkaline attack was carried out to determine the reactive fraction of Slag A and Slag R, in terms of Al and Si ion release. In particular, the interest is in the reactive Si/Al mass ratio calculated as ratio of the amount of Si and Al measured in the eluates. This ratio is considered a reactive ratio because it takes into account only the amount of Si and Al dissolved in the alkaline solution and not the total amount of these ions in the starting materials. It has been observed by the authors [28,29] for other kind of slag that the amorphous fraction is the more reactive with respect to the crystalline type. Confirming previous results, Slag A, with an amorphous fraction of about 56 wt.% presented higher contents of soluble Si and Al species in the eluate (Table 4) occurrence that is directly related to the higher amorphous fraction than the Slag R (35 wt.%). This behavior can also be correlated to the higher specific surface of Slag A (7.61 m^2^/g) with respect to Slag R (3.52 m^2^/g), reported in Česnovar et al. [21] which favors the slag reactivity and dissolution. Being the amorphous fraction of the slags, the most reactive fraction in alkali solution, the Si/Al mass ratio appears to be critical for the degree of reticulation of the final alkali-activated solid material. When the Si/Al mass ratio is below the value of 3 the corresponding materials are characterized by a 3D rigid network, suitable for a concrete, cement, or waste-encapsulating medium [28,31]. In the present study, the Si/Al mass ratio in alkali solution was similar for both slags, thus indicating that both slags could produce AAM with good mechanical performances even though the amount of SiO_2_ and Al_2_O_3_ differs for the two slags.

The alkaline activation induced an extremely alkaline environment, so the proposed basic approach has proved to be useful to study the chemical stability of minor components of the slags that generally have a high environmental impact: the heavy metals. In addition, amphoteric elements, such as Sb, As, and Mo, were extremely interesting to investigate because they can be easily leached out in NaOH solution. The concentration of metals in the eluate is reported in Table 4. From these values it appears evident that the releases of heavy metals are higher with respect to those in aqueous environment (see Table 3). This behavior can be related to: (i) the strong conditions of the test in NaOH where the solution is at 80 °C with a concentration of 8M, and (ii) the different chemical behavior of each element. Among heavy metals, Ba and Zn are also the most released elements in water—even though in lower amount—while all the other elements are released in alkaline environment only.

**Figure 4:**
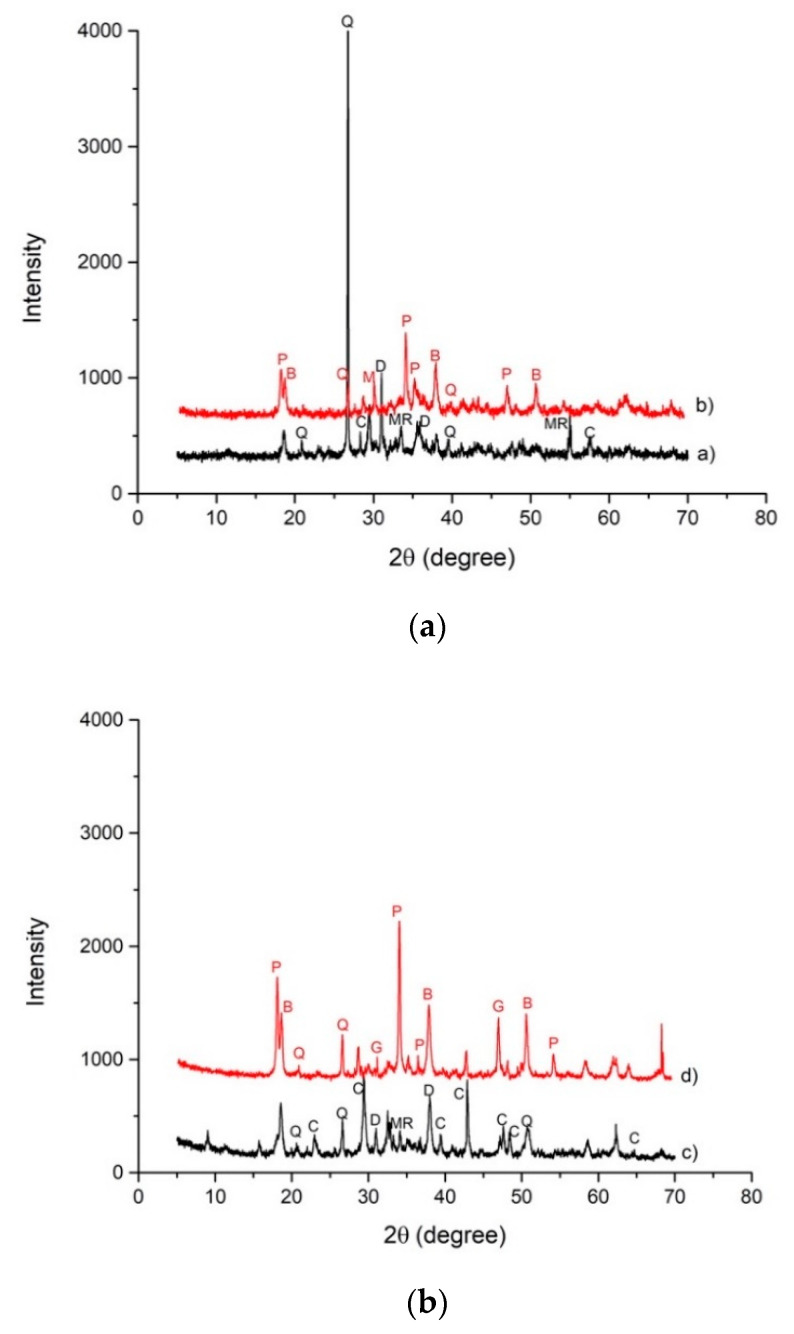
(**a**) XRD patterns collected on Slag A a) and Slag A after NaOH 8M test b) (C = calcite, P = portlandite, Q = quartz, D = dolomite, MR = merwinite, B = brucite). (**b**) XRD patterns collected on Slag R c) and Slag R after NaOH 8M test d) (C = calcite, P = portlandite, Q = quartz, MR = merwinite, B = brucite, G = gehlenite).

Mineralogical analysis was performed in order to estimate the modifications in the crystalline phases after the alkaline attack (Figure 4). Slag A was mainly characterized by Q-quartz (SiO_2_), C-calcite (CaCO_3_), and D-dolomite CaMg(CO_3_)_2_ and merwinite Ca_3_Mg(SiO_4_)_2_. After the NaOH, 8M attack, a significant decrease of quartz (Q), was recorded while calcite (C), dolomite (D), and merwinite (MR) disappeared. The alkaline attack formed new phases: portlandite (Ca(OH)_2_) (P), brucite (Mg(OH)_2_ (B), and magnetite (Fe_3_O_4_) (M) (Figure 4a). Main crystalline phases identified in Slag R were: Q-quartz (SiO_2_) and C-calcite (CaCO_3_) and D-dolomite CaMg(CO_3_)_2_. The last two have higher intensity with respect to Slag A as evidenced both by Rietveld analysis, reported in Table 2, and by higher amounts of Ca and Mg in chemical analysis (Table 1). After the alkaline attack, a significant decrease of quartz (Q), was recorded while calcite (C) and dolomite (D) disappeared, and the formation of portlandite (Ca(OH)_2_) (P), brucite (Mg(OH)_2_ (B), magnetite (Fe_3_O_4_) (M), and gehlenite (Ca_2_Al_2_SiO_7_) (G) occurred (Figure 4b). Formation of portlandite is particularly evident in Slag R where the content of Ca is higher with respect to Slag A. From XRD pattern appears that even if some phases decrease in intensity and others form, in general, crystalline phases present in the as-received slags remain almost undissolved after NaOH dissolution.

To evaluate the behavior of the slags in acid environment the HCl attack was performed. This test allows quantification of the phases formed as a result of alkaline activation, thus measuring the reactive capacity of the slag to form a geopolymer network in alkaline environment. For this reason, the test was performed on the slags before and after alkaline activation to compare such behavior.

**Figure 5:**
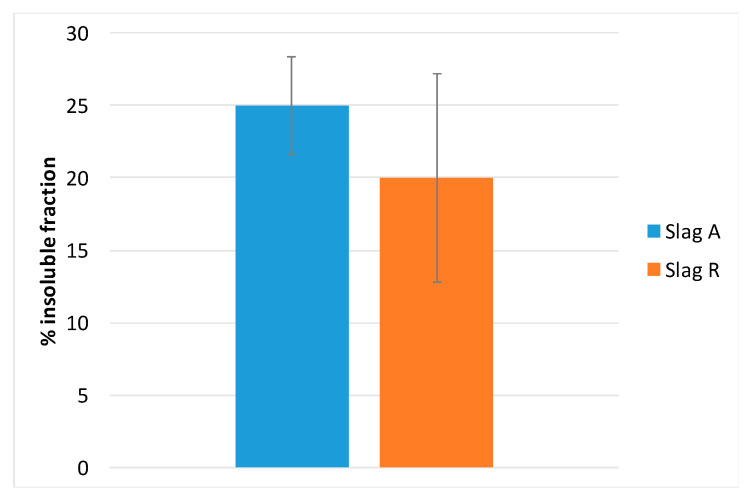
Insoluble fraction after HCl test of Slag A and Slag R.

**Figure 6:**
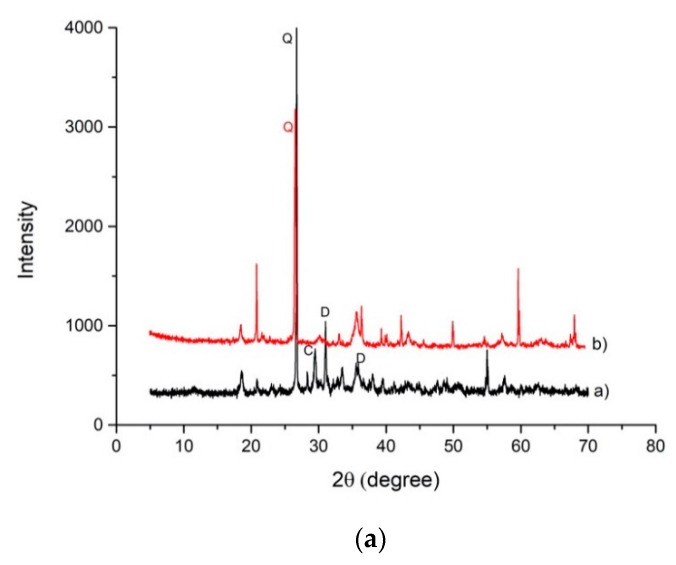

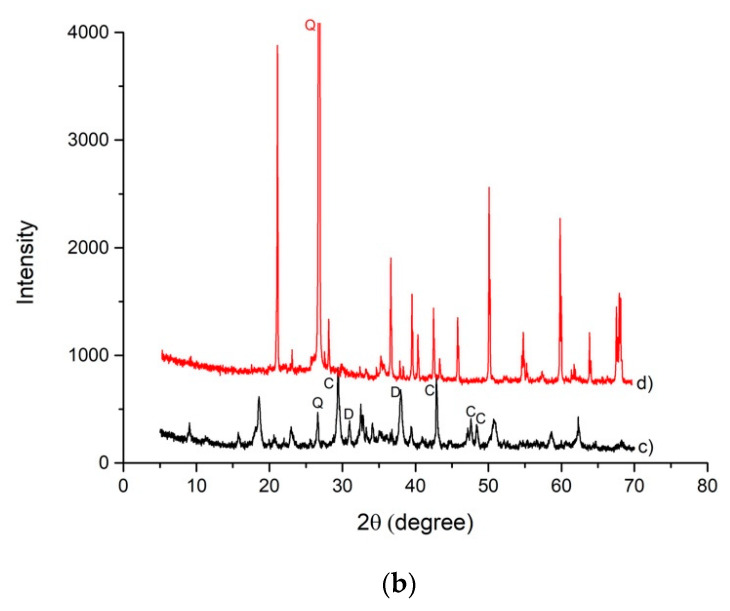
(**a**) XRD patterns of Slag A a) and Slag A after acid attack b) (C = calcite, Q = quartz, D = dolomite). (**b**) XRD patterns of Slag R c) and Slag R after the acid attack d) (C = calcite, Q = quartz, D = dolomite).

The insoluble fraction of Slag R was lower than the insoluble fraction of Slag A because the first reacted more than the second (Figure 5). Slag R has a calcium content of about 13 wt.% and it was characterized by L.O.I. of about 20 wt.%, both these parameters justified by the presence of calcite and dolomite that were responsible of its high solubility in hydrochloric acid (carbonates content is higher than 30%). In contrast, Slag A has a calcium content of about 7 wt.% and L.O.I. of 14 wt.%, thus a lower solubility in HCl was recorded. The insoluble fraction of both slags was analyzed by XRD; the data indicate that only quartz remained in the insoluble phase while calcite and dolomite were dissolved during HCl test (Figure 6a,b). Slag A and Slag R were characterized by the same behavior from mineralogical point of view after acid attack because of the similarity of the crystalline phases present, the difference is only related to carbonates amount.

### 3.2. Alkali Activated Materials Characterization

In order to estimate the consolidation of the structure of the alkali-activated materials, a sample of each AAMs formulation, A50NW and R50NW, was immersed in distilled water to perform an evaluation of the structural integrity of the samples. In order to qualitatively evaluate the efficacy of the consolidation process, the samples were observed by naked eye to see if they alter their aspect in this situation. Neither sample dissolves in water confirming the occurrence of alkali activation reaction.

This integrity test has been adopted in our laboratory as a common procedure to evidence the chemical stability of alkali-activated materials containing complex aluminosilicate powders with variable chemical and mineralogical compositions, such as incinerator bottom ash [27] and mine tailings [23].

**Figure 7:**
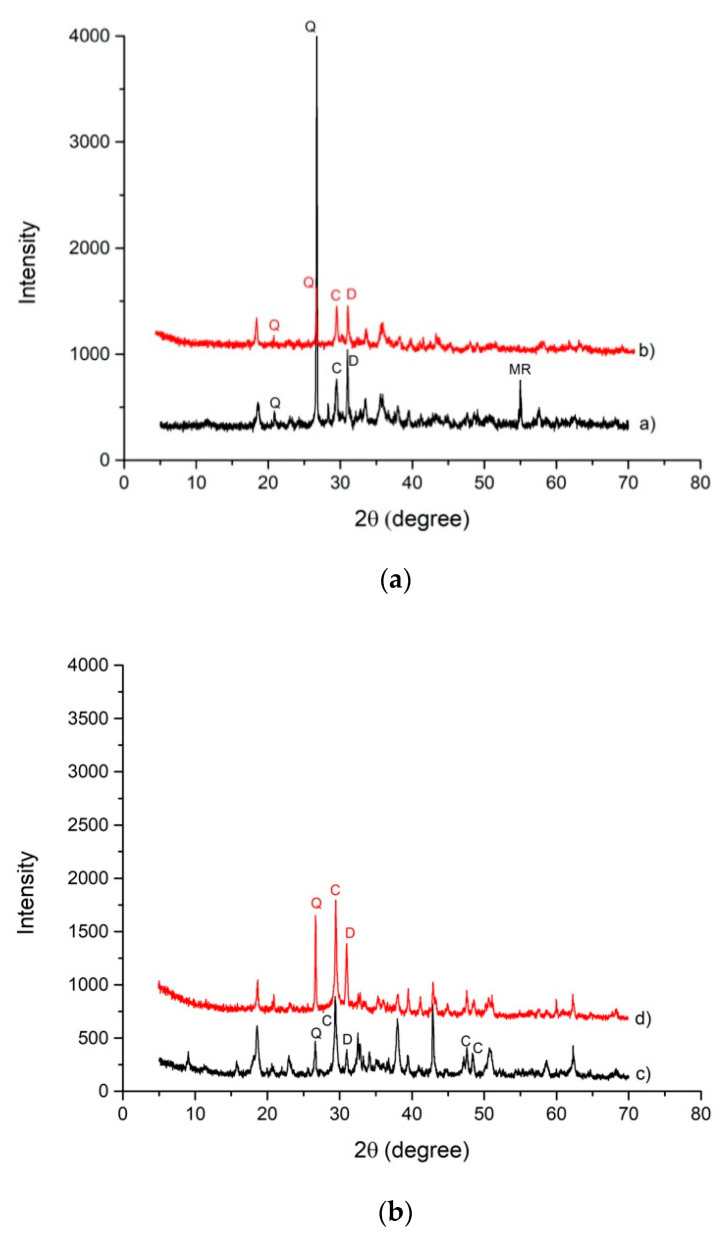
(**a**) XRD patterns of Slag A a) and A50NW AAM b) (C = calcite, Q = quartz, D = dolomite, MR = merwinite). (**b**) XRD patterns of Slag R c) and R50NW AAM d) (C = calcite, Q = quartz, D = dolomite).

The comparison of XRD patterns between slag A and R and their respective alkali-activated materials (Figure 7a,b) show the presence of all minerals obtained from each precursor, but in a different quantity compared to the original amount. Alkaline activation products, such as amorphous calcium silicate hydrate gel C–S–H, are formed as part of the reaction between the activator and Ca^2+^, abundant in the precursors, especially in slag R [30,32,33].

As discussed above, since the alkaline environment can modify the solubility of some elements, such as Cr, Ni, As, Cd, a leaching test in water (according to EN 12457-2) on the alkali-activated materials was necessary to verify the eventual hazardous nature. The formation of the 3D network of Ca-Na-aluminosilicate as a consequence of the consolidation represents an alternation of silicon and aluminum atoms in four-fold coordination with oxygen. Being the electric charge of Al +3, with respect to +4 for Si, the AlO_4_ tetrahedron is charged −1, thus attracting positive metallic cations, within the geopolymer network. If the number of AlO_4_^−1^ in the 3D geopolymer network is high, the chemical stability of the bonded cations is high as well, thus the amount of metallic cations in the eluate will be low [34].

The amount of heavy metals found in the eluate of A50NW and R50NW was compared to the limit values prescribed to dispose of non-dangerous waste in a landfill (same as indicated above for the slags) (Table 5). All the release values are below the legal limit. An interesting comparison is the release values of slags before alkali activation in both the environments, NaOH and water. All the bivalent ions (Ba, Cd, Cu, Ni, Pb, and Zn) are well immobilized in a geopolymeric matrix. In particular, for Cd and Pb no release is shown. On the contrary, amphoteric elements, such as As and Cr, show a slight increase of release with respect to the corresponding slag in both the environments. These results confirm the findings of the authors [27] for immobilization of incinerator fly ashes in metakaolin-based geopolymers, where for bivalent cations very low release are observed. In particular, for Cd, negligible release as found, as already reported by Izquierdo et al. [35] and Zhang et al. [36], who explained this behavior by considering the formation of cadmium hydroxide responsible for the immobilization of Cd within the geopolymer.

Particular attention was paid to the release of amphoteric elements Mo, Sb, and As before, and after alkaline activation of both slags (Table 6). Specifically, the results related to the test on alkali-activated materials derive from leaching in water, while the values of the two slags derive from NaOH test. Since after few minutes in water the pH of the leaching medium becomes alkaline because of the nature of AAMs, these experimental results are comparable. These results show how the release of Sb and As of AAM slightly increases with respect to the corresponding slag, still remaining below the legal limits [37,38,39].

On the other hand, in the AMMs specimens the Mo presents an increase of leaching values that slightly exceeds the legal limit for landfill for non-dangerous waste.

These results are in line with literature [39] showing good retention of Pb, Cd, Cr, Cu, Ni, Zn, even when the leachable contaminants content was measured by conducting compliance leaching tests according to EPA Test Method 1311 (toxicity characteristic leaching procedure-TCLP) [40].

**Figure 8:**
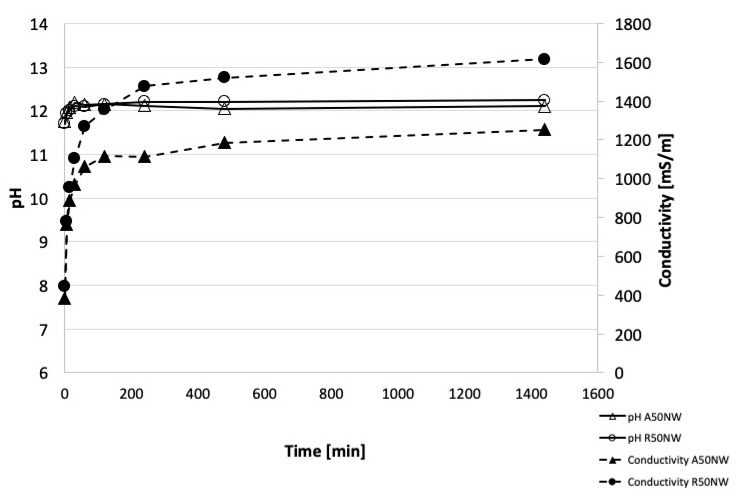
pH values of A50NW and R50NW AAMs (lines are drawn for eye help, only). The confidence interval for pH is about 1%, while for conductivity is around 2%.

The pH and ionic conductivity measurements were additional experimental measures to assess the reticulation process and the three dimensional reticulation extent. The alkali-activated material A50NW obtained from Slag A presented a very stable pH values and low values of ionic conductivity (from 385 to 1251 mS/m), indicating a low amount of ions released from the geopolymeric network (Figure 8). The stable pH values indicated that the excess of OH^−^ not reacted with the aluminosilicate powders was released in the very first seconds of the contact between the solid and water. Subsequently, no other OH^−^ groups were released. R50NW alkali-activated material was characterized by a similar behavior of pH values recording values almost constant (11.7–12.2) while conductivity slightly increased (from 445 to 1615 mS/m). The A50NW presented a slightly higher chemical stability which confirmed the more efficient reticulation of the structure with respect to R50NW (Figure 8). This behavior confirms the hypothesis noted above that slag with higher amorphous fraction also shows higher reactivity in alkaline environment.

As conclusion, we can state that these values of pH and ionic conductivity are typical for alkali-activated materials, with good reticulation and almost complete reaction of the alkaline solution added for activation. Similar trends are already reported in literature for geopolymers based on metakaolin [41] and alkali-activated materials from metakaolin added with residues [42].

Testing in HCl was performed to quantify the phases formed as a result of alkaline activation and to establish the reactive capacity of slag in alkaline environment with the formation of a geopolymer. The main reaction product formed in this process is an amorphous/semicrystalline aluminosilicate gel. After the test, the insoluble fraction corresponds to the part that had not reacted with the alkaline solutions because HCl provokes the dissolution of the reaction products formed during alkali activation of slag as indicated in previous study [43].

**Figure 9:**
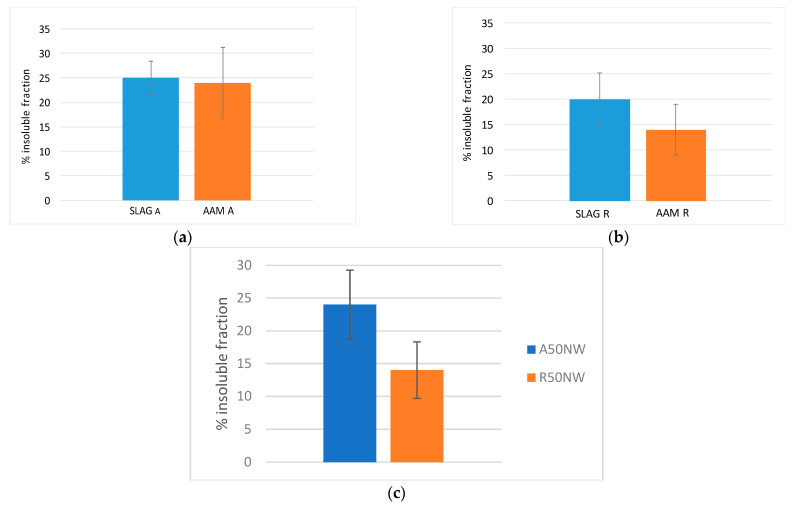
Comparison between Slag and corresponding AAM after test in HCl for Slag A (**a**) and R (**b**); (**c**) insoluble fraction of A50NW and R50NW AAMs after HCl test.

The results of the test on alkali-activated materials containing A and R slags show that the insoluble fraction of R50NW was less than the insoluble residue of A50NW (Figure 9c). It confirmed the results of the same test on the slags. The results are distorted by the presence of carbonatic crystalline phases in the alkali-activated materials-carbonates that almost did not react during the activation. Yet, these phases are soluble in HCl test, so the soluble fraction sums up the percent of carbonates and the reaction products formed during activation. This is also confirmed by the comparison between slags and the corresponding AAMs after test in HCl. Slag A (Figure 9a) shows lower solubility, both as in the as-received state and after alkali activation, with respect to Slag R, and corresponding AAM (Figure 9b). This fact is related to the lower carbonatic content. For Slag R, the presence of unreacted fraction of carbonates, in the slag as well as in the corresponding AAM, leads to higher solubility. The aluminosilicate fraction created during the alkaline activation process is less soluble with respect to original calcite and dolomite. For AAM with high carbonatic content, this test needs a different discussion with respect to materials that do not contain carbonates.

**Figure 10:**
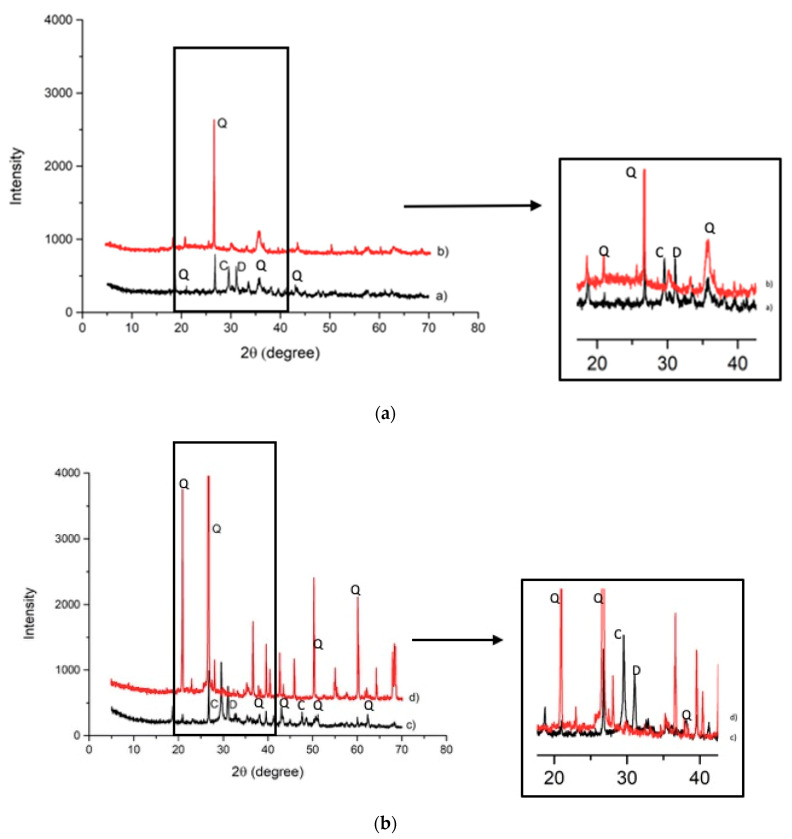
(**a**) XRD pattern of A50NW AAM a), A50NW AAM after HCl test b) (C = calcite, D = dolomite, Q = quartz). (**b**) XRD pattern of R50NW AAM c), R50NW AAM after HCl test d) (C = calcite, D = dolomite, Q = quartz).

The insoluble residue resulting from the HCl test was analyzed by XRD to evaluate the modifications in crystalline phases after the acid attack. X-ray analysis showed that only quartz remained. The calcite and dolomite were dissolved during HCl test (Figure 10a,b), contributing to the amount of soluble fraction. Both alkali-activated materials were characterized by the same behavior of their respective slags. XRD patterns shows the presence of broad band between 20 and 40° in 2θ typical of an amorphous fraction that has not been dissolved in HCl, hence it is composed of Al-O-Si network. This behavior is particularly evident in AAM containing slag A, which is richer in amorphous phase (56%) with respect to slag R (35%). Such glassy fraction is more reactive in alkaline environment forming C–(N–)A–S–H–gel, as already observed by Adesanya et al. [16] and more insoluble in HCl acid with respect to carbonatic fraction. Carbonatic fraction in AAM plays the role of inert phase and do not contribute to alkali activation. The formation of C–(N–)A–S–H–gel is confirmed by EDS analysis which shows that the chemical composition of the gels formed in the two alkali-activated materials is Na = 7.7, Ca = 10.4, Al = 2.3, and Si = 11.2 for A50NW and Na = 8.3, Ca = 8.3, Al = 1.4, and Si = 10.2 for R50NW (Figure 2). The more significant difference is related to Ca content, because notwithstanding the higher amount of Ca in slag R, it corresponds to crystalline phases insoluble to alkali activation and therefore not included in amorphous gel.

## 4. Conclusions

In the present study, the electric arc furnace carbon or stainless steel slag (EAF) and secondary metallurgical slag such as ladle furnace basic slag (LS) were used as precursors in alkali-activation process to obtain chemically stable solid manufacts. Their chemical and microstructural performance was analyzed by XRD and disruption of structural units in water and acidic media was checked via chemical analysis of the leached ions.

Slag A showed lower pH and conductivity with respect to Slag R, indicating that slag A was more stable and less soluble in an aqueous environment than Slag R. Slag A, with its amorphous fraction of about 56%, presented higher contents of soluble Si and Al species in the eluate occurrence that is directly related to the higher amorphous fraction with respect to Slag R (35%). The soluble Si/Al mass ratio in alkali solution was similar for both slags, thus indicating that both slags are suitable to produce AAM, even though the amount of SiO_2_ and Al_2_O_3_ differs between the two slags. Considering the leaching tendency of heavy metals, Ba and Zn are the most released elements in water-even though in lower amount, while all the other elements are released in an alkaline environment only.

In order to qualitatively evaluate the effectiveness of the consolidation process, the AAMs samples were subjected to integrity tests in water. Neither sample dissolved, confirming the occurrence of a sufficiently extended alkali activation reaction. The leaching behavior of AAMs shows that all the release values are below the legal limit. An interesting comparison is with the release values of slags before alkali activation in both the environments, NaOH and water. All the bivalent ions (Ba, Cd, Cu, Ni, Pb, and Zn) are well immobilized in a geopolymeric matrix. In particular, for Cd and Pb no release is shown. On the contrary, amphoteric elements such as As and Cr, show a slight increase of release with respect to the corresponding slag in both the environments. A particular attention was paid to the release of amphoteric elements Mo, Sb, and As before and after alkaline activation of both slags. These results show how the release of Sb and As of AAM slightly increases with respect to the corresponding slag, still remaining below the legal limits. On the other hand, in the AMMs specimens the Mo presents an increase of leaching values that slightly exceed the legal limit for landfill for non-dangerous waste. 

pH and ionic conductivity results are in the range of metakaolin-based geopolymers and other alkali-activated materials, indicating good reticulation and complete reaction of the alkaline solution added for activation.

The results of the test in HCl on AAMs-containing slags show that the insoluble fraction of R-slag-based samples was lower than the insoluble residue of A containing AAM. It confirms the results of the same test on the slags. These data are distorted by the presence of carbonatic crystalline phases in the alkali-activated materials, carbonates that almost did not react during the alkali activation. Further, these phases are soluble in HCl test, so the soluble fraction sums up the percent of carbonates and the reaction products formed during activation.

## Figures and Tables

**Table 1:** Chemical composition of Slag A and Slag R, (**a**) main and (**b**) other elements.

Oxides (wt.%)	Slag A	St.Dev	Slag R	St.Dev
(**a**)				
SiO_2_	21.05	0.02	13.80	0.15
Al_2_O_3_	8.54	0.09	5.25	0.07
Fe_2_O_3_	11.37	0.08	4.69	0.07
CaO	20.87	0.26	28.10	0.34
MgO	14.88	0.32	23.44	0.27
Na_2_O	0.13	0.01	0.30	0.03
K_2_O	0.18	0.01	0.15	0.01
Cr_2_O_3_	3.76	0.02	0.19	0.01
MnO	2.24	0.03	0.62	0.01
LOI	14.15	0.01	20.47	0.01
OTH	1.3	/	2.1	/
(**b**)				
P_2_O_5_	0.122	0.009	0.08	0.01
SO_3_	0.23	0.02	1.229	0.006
TiO_2_	0.40	0.02	0.190	0.003
V_2_O_5_	0.0734	0.0002	0.032	0.003
Co_3_O_4_	0.010	0.002	<L.Q.	/
NiO	0.1017	0.0006	0.026	0.005
CuO	0.026	0.001	0.010	0.001
ZnO	0.083	0.002	0.2	0.3
As_2_O_3_	0.03	0.01	0.029	0.002
SrO	0.0297	0.0005	0.029	0.0001
BaO	0.03	0.01	0.024	0.009
PbO	0.017	0.003	0.008	0.003

LOI—loss on ignition at 950 °C; OTH—sum of other components

**Table 2:** Quantitative mineralogical analyses of Slag A and Slag R (all in wt.%). Rietveld refinement was performed by X’Pert High Score Plus diffraction software with goodness of fit of 4.9.

Mineralogical Phase	Slag A	Slag R
Quartz 00-046-1045 SiO_2_	6.5	12.9
Wuestite FeO	0.7	0.1
Dolomite MgCa(CO_3_)_2_	8.6	19.4
Chromite Cr_2_O_3_	6.8	0.1
Calcite CaCO_3_	7.2	13.2
Ankerite Ca(fe,Mg,Mn)(CO_3_)_2_	0.3	2.1
Corundum Al_2_O_3_	1.5	1.2
Merwinite Ca_3_Mg(SiO_4_)_2_	8.8	4.5
Periclase MgO	3.1	6.9
Gehlenite Ca_2_Al(AlSi)O_7_	0.9	/
Mayenite Ca_12_Al_14_O_33_	/	0.4
Larnite Ca_2_SiO_4_	/	3.9
Brucite Mg(OH)_2_	/	0.2
Amorphous	55.6	35.0

**Table 3:** Heavy metals (mg/L) in the slag A/R eluates after leaching test in water (EN 12457-2).

Heavy Metals (mg/L)	Slag A	Slag R	Law Limit
As	<L.Q.	<L.Q.	0.2
Ba	1.13 ± 0.34	1.1 ± 0.33	10
Cd	<L.Q.	<L.Q.	0.1
Cr	<L.Q.	<L.Q.	1
Cu	0.26 ± 0.08	<L.Q.	5
Hg	<L.Q.	<L.Q.	0.02
Ni	<L.Q.	<L.Q.	1
Pb	<L.Q.	<L.Q.	1
Zn	0.92 ± 0.28	0.59 ± 0.18	5

**Table 4:** ICP-OES determination of the soluble Si and Al atoms, heavy metals, and amphoteric elements in NaOH 8M solution, at 80 °C.

Element (mg/L)	Slag A	Slag R
Al	127 ± 38	78 ± 23
Si	236±71	142 ± 43
Si/Al	2.08	1.98
As	0.069 ± 0.021	0.104 ± 0.031
Ba	1.612 ± 0.484	0.573 ± 0.172
Cd	<L.Q.	<L.Q.
Cr	0.021 ± 0.007	0.034 ± 0.01
Cu	0.145 ± 0.044	0.117 ± 0.035
Ni	0.044 ± 0.013	0.011 ± 0.003
Pb	0.418 ± 0.125	0.165 ± 0.049
Zn	4.0 ± 1.2	10 ± 3
Mo	0.487 ± 0.146	0.104 ± 0.031
Sb	0.020 ± 0.006	0.012 ± 0.004

**Table 5:** Heavy metals (mg/L) in A50NW and R50NW AAMs eluates after leaching test (EN 12457-2).

Heavy Metals (mg/L)	A50NW	R50NW	Law Limit
As	0.097 ± 0.029	0.163 ± 0.049	0.2
Ba	0.017 ± 0.005	0.018 ± 0.005	10
Cd	<L.Q.	<L.Q.	0.1
Cr	1.12 ± 0.336	0.196 ± 0.059	1
Cu	0.06 ± 0.018	0.04 ± 0.012	5
Ni	0.02 ± 0.006	0.061 ± 0.018	1
Pb	0.013 ± 0.004	<L.Q.	1
Zn	0.079 ± 0.024	0.075 ± 0.023	5

**Table 6:** Amphoteric elements (mg/L) released before and after alkali activation.

Leaching in Distilled Water	A50NW	R50NW	Law Limit
Mo	1.477 ± 0.443	1.004 ± 0.301	1
Sb	0.018 ± 0.005	0.021 ± 0.006	0.07
As	0.097 ± 0.029	0.163 ± 0.049	0.2
**Leaching in NaOH**	**Slag A**	**Slag R**	**Law Limit**
Mo	0.487 ± 0.146	0.104 ± 0.031	1
Sb	0.020 ± 0.006	0.012 ± 0.004	0.07
As	0.069 ± 0.021	0.104 ± 0.031	0.2

## Data Availability

The data presented in this study are available on request from the corresponding author.
